# Never-resting microglia: physiological roles in the healthy brain and pathological implications

**DOI:** 10.3389/fncel.2014.00240

**Published:** 2014-08-15

**Authors:** Amanda Sierra, Marie-Ève Tremblay, Hiroaki Wake

**Affiliations:** ^1^Ikerbasque Foundation, University of the Basque Country EHU/UPVBilbao, Spain; ^2^Axe Neurosciences, Centre de Recherche du CHU de Québec, Université LavalQuébec, Canada; ^3^Division of Brain Circuits, National Institute for Basic Biology, NINSOkazaki, Japan

**Keywords:** microglia, health, disease, phenotype, neuroprotection, phagocytosis, synapses, neurogenesis

Microglia are the resident macrophages of the central nervous system (CNS), largely known as the major orchestrators of the immune response. As such, they have been traditionally studied in various contexts of trauma, injury and disease, where their phenotypic transformation (or “activation”) has been assumed to induce a wide range of mostly detrimental effects. In the last few years, however, a series of discoveries have started to unravel their active and positive contribution to normal CNS function.

Within this perspective, our research topic *Never-resting microglia: physiological roles in the healthy brain and pathological implications* reviews the emerging roles of microglia in the healthy CNS across development, adulthood, and normal aging. The cellular and molecular mechanisms underlying these new roles are particularly covered, such as microglial phagocytosis and dynamic interactions with synapses, as much as their functional consequences for the regulation of neuronal circuit maturation and refinement, activity, and plasticity, including adult hippocampal neurogenesis.

Since these findings provide a better understanding of microglial function in health as much as in disease, our research topic also summarizes the current view about microglial origin, homeostasis, diversity, phenotypic transformation, neurotoxicity, relationships with macrophages from the periphery, and contribution to normal CNS aging and various pathological conditions. Additionally, novel methodological approaches and molecular tools to study microglia in their normally prevailing state are discussed.

In particular, Ginhoux et al. (2013) review the history of microglial cells and discuss the latest advances in our understanding of their origin, differentiation, and homeostasis, thus providing new insights into their roles in health and disease.

Wolf et al. (2013) summarize the recent efforts to exploit CX3CR1 promoter activity for the visualization and genetic manipulation of microglia, in order to probe their functional contributions in the CNS, and the resulting insights into the role of CX3CR1.

Karperien et al. (2013) review current trends and methods of fractal analysis, used for quantitating changes in microglial morphology and differentiate subtle differences amongst ramified cells, while focusing on box counting analysis, including lacunarity and multifractal analysis.

Hanisch (2013) discuss the diversity in microglial cells protein expression, housekeeping functions, and reactive phenotypes, which could result from differences in lineage commitment and microenvironment, or stochastic variation.

Kierdorf and Prinz (2013) summarize current knowledge of the intrinsic (e.g., Runx-1, Irf8, Pu.1) and extrinsic factors (e.g., CD200, CX3CR1, TREM2) which regulate the transition from a surveying microglial phenotype to an activated stage.

Hellwig et al. (2013) critically reconsider the term microglial neurotoxicity and discuss experimental problems around microglial biology (e.g., *in vitro* preparations and transgenic strategies) which often have led to the conclusion that microglia are neurotoxic cells.

London et al. (2013) discuss the functional heterogeneity and relationships between microglia and bone-marrow derived macrophages, their contribution to CNS plasticity and repair, and the lessons derived from other populations of tissue-resident macrophages.

Sierra et al. (2013) summarize the current state of the literature regarding the role of microglial phagocytosis in maintaining tissue homeostasis in health as in disease, and the underlying molecular mechanisms including find-me, eat-me, and digest-me signals.

Miyamoto et al. (2013) focus on the interactions between microglia and synapses, reviewing the cellular and molecular mechanisms mediating their contacts, and their possible implications in the fine tuning of neural circuits during normal physiological conditions.

Domercq et al. (2013) summarize the relevant data regarding the role of neurotransmitter receptors in microglial physiology and pathology, with an emphasis on purinergic and glutamate receptors which modulate microglial physiology in various manners.

Béchade et al. (2013) discuss the role of microglia in the control of neuronal activity, describing how their dysfunction is responsible for the alteration of neuronal activity in pathological situations, and how microglia can be considered as partners of neurotransmission in the healthy brain.

Gemma and Bachstetter (2013) review the role of microglia in hippocampal neurogenesis during normal physiological conditions, with an emphasis on microglial phagocytosis, release of trophic factors, and the involvement of CX3CR1.

Belarbi and Rosi (2013) summarize the current knowledge on how the production, distribution, and recruitment of new neurons into behaviorally relevant neural networks are modified in the inflamed hippocampus.

Laslty, Wong (2013) explore the hypothesis that age-related changes in microglia could be implicated in the pathogenic mechanisms of age-related neurodegenerative diseases, discussing the possible underlying cellular mechanisms, as well as “rejuvenative” measures and strategies.

In conclusion, this special issue seeks to emphasize how the current research in neuroscience is being challenged by never-resting microglia.

## Conflict of interest statement

The authors declare that the research was conducted in the absence of any commercial or financial relationships that could be construed as a potential conflict of interest.
